# Socioeconomic status and education level are associated with dyslipidemia in adults not taking lipid-lowering medication: a population-based study

**DOI:** 10.1093/inthealth/ihz089

**Published:** 2019-11-06

**Authors:** Luçandra R Espírito Santo, Thaís O Faria, Carla Silvana O Silva, Lorena A Xavier, Vivianne C Reis, Gabriel A Mota, Marise F Silveira, José Geraldo Mill, Marcelo P Baldo

**Affiliations:** 1 Department of Medicine, Montes Claros State University, Montes Claros, MG 39401-089, Brazil; 2 Department of Nursing, Prominas University, Montes Claros, MG 39401-089, Brazil; 3 Department of Nursing, Montes Claros State University, Montes Claros, MG 39401-089, Brazil; 4 Department of Physical Education, Montes Claros State University, Montes Claros, MG 39401-089, Brazil; 5 Department of Statistics, Montes Claros State University, Montes Claros, MG 39401-089, Brazil; 6 Department of Physiological Sciences, Federal University of Espírito Santo, Vitória, ES 27042-755, Brazil; 7 Department of Medicine, Centro Universitário, UniFIPMOC, Montes Claros, MG 39408-007, Brazil; 8 Department of Pathophysiology, Montes Claros State University, Montes Claros, MG 39401-089, Brazil

**Keywords:** dyslipidemia, education level, lipid profile, socio-economic status

## Abstract

**Background:**

Socio-economic disparities account for changes in the lipid profile in developing countries. We aimed to investigate the association between blood lipids and socio-economic and educational strata in adults not taking lipid-lowering medications.

**Methods:**

A cross-sectional, population-based study enrolled 1614 individuals not taking lipid-lowering medications. Sociodemographic characteristics, monthly income, education level and the number of consumer goods available at home were obtained and individuals were classified into five socio-economic categories. Blood lipids were obtained in fasting participants.

**Results:**

In men, the higher the socio-economic or educational stratum, the higher the total cholesterol, low-density lipoprotein cholesterol (LDL-c) and triglyceride (TG) levels and the lower the high-density lipoprotein cholesterol (HDL-c), after controlling for age, body mass index, hypertension, smoking habit and physical activity. In women, the higher socio-economic strata were associated with elevated total cholesterol and HDL-c, while lower total cholesterol, LDL-c and TG levels were found in those with higher education levels. Also, individuals in the upper socio-economic strata had higher levels of total cholesterol and LDL-c, showing more than two times higher odds of having multiple alterations in blood lipids (men: OR 2.99 [95% CI 1.23 to 5.07]; women: OR 2.31 [95% CI 1.09 to 5.83]).

**Conclusions:**

Dyslipidemia is highly prevalent in developing countries. Individuals in the highest socio-economic category are the ones at higher risk for dyslipidemia. This phenomenon calls for strategies to stimulate healthy diet habits and a physically active lifestyle to minimize health problems.

## Introduction

Even in the face of the major therapeutic advances observed in recent decades, cardiovascular diseases (CVDs) figure as the main cause of death among men and women.^1,2^ Recent data show CVDs are the leading causes of death in the USA, with coronary heart diseases representing 43% of the total deaths attributed to CVDs.^2^ In Brazil, approximately 30% of annual deaths are due to CVDs. According to Brazilian registry data, 335 177 people died of CVDs in 2011, 103 475 deaths were associated with ischemic heart diseases and 100 470 due to cerebrovascular diseases.^1,3^

Several risk factors are associated with the development of CVDs, including smoking, abusive alcohol consumption, sedentary lifestyle and poor nutrition.^4^ In fact, the main risk factor for an acute coronary event is dyslipidemia, as high serum levels of cholesterol are the main factor.^5,6^ A study conducted by Martinez et al.^7^ showed that 40% of Brazilians have higher than recommended cholesterol levels, which have been associated with high cardiovascular morbidity and mortality.

Dyslipidemia, as with other complex multifactorial diseases, emerges from the interaction of a genetic background with environmental and socio-economic factors. Despite the biological determinants, important epidemiological studies have shown that some social predictors, such as socio-economic stratum and education level, are also associated with changes in the lipid profile and thus influence the development of CVDs. In developed countries, cholesterol levels decrease with the progression to higher socio-economic or education levels, mainly in women.^5,8,9^ However, some characteristics observed in developing countries show a different pattern, in which high serum cholesterol is found in the highest socio-economic stratum.^10,11^

The Brazilian population has great disparities regarding education and socio-economic levels. However, there is a lack of population-based studies evaluating the association between socio-economic and education levels with the lipid profile in developing countries. Also, some studies have used education level as synonymous with socio-economic status,^12^ even though socio-economic status is established based on several other variables in which education level is included. Thus we aimed to investigate the association between blood levels of total cholesterol, high-density lipoprotein cholesterol (HDL-c), low-density lipoprotein cholesterol (LDL-c) and triglycerides (TG) with the different socio-economic and educational strata in the general population not taking lipid-lowering medications.

## Materials and methods

### Study population

The Monitoring of Trends and Determinants in Cardiovascular Disease (MONICA) Vitória is a population-based, cross-sectional study that includes the urban adult population (25–64 y) in Vitória, Brazil. The project was designed to determine the prevalence of cardiovascular risk factors and its association with cardiovascular diseases. The data were collected according to the WHO MONICA Project guidelines, and the design and sampling of this study have been published in more detail in previous publications.^13,14^ A group of 1662 individuals was selected based on a multistage probability sample and arrived at the University Hospital for clinical and laboratory examination. All data were collected on a single prescheduled day in the morning. The project is in accordance with the ethical standards of the Helsinki Declaration of the World Medical Association and was approved by the institutional ethics committee, and all participants gave written informed consent.

### Clinical and biochemical evaluation

Blood pressure (BP) was measured in fasting individuals, in the left arm, kept in a sitting position and after a rest period of 5–10 min, using a mercury column sphygmomanometer. The first and fifth Korotkoff phases were used to indicate systolic BP (SBP) and diastolic BP (DBP), respectively. Heart rate was calculated by counting pulse beats during 30 s.

Anthropometric parameters were obtained using standard methods^15^ and collected by trained technicians. Body weight was obtained on a calibrated scale with an accuracy of 0.1 kg. Height was measured on a wall stadiometer with an accuracy of 0.5 cm. The body mass index (BMI) was measured as the ratio of body weight (kg) to height squared (m^2^). Overweight was defined as a BMI of 25–29.9 kg/m^2^ and obese was defined as a BMI ≥30 kg/m^2^. Waist circumference was measured at the midpoint between the last costal arch and the iliac crest, considering the maximum point of normal expiration, with the individual in the standing position. The hip circumference was measured with an accuracy of 0.1 cm around the thighs, at the height of the greater trochanter, with the individual standing.

Biochemical blood parameters were obtained in participants instructed to fast for at least 10 h before the exam. The blood samples were drawn after venipuncture in the upper limb, performed by a previously trained laboratory technician. The samples were sent to the central laboratory (SESI, Vitória, Brazil) for biochemical analysis using commercially available kits. The fasting glucose level was measured from blood collected in a tube containing fluoride as anticoagulant, and diabetes was defined as having a fasting blood glucose ≥126 mg/dL. For the lipids dosage, the anticoagulant used was ethylenediaminetetraacetic acid. The LDL-c fraction was calculated indirectly by the Friedewald equation for TG <400 mg/dL. Dyslipidemia was considered to be present with one or more of the following conditions, following the Brazilian Guidelines for Dyslipidemias:^16^ total cholesterol ≥200 mg/dL, LDL-c ≥160 mg/dL, TG ≥150 mg/dL and HDL-c <40 mg/dL in men and <50 mg/dL in women. For the purpose of the present study, we removed those individuals who were on any lipid-lowering medication or on a cholesterol-restricted diet (48 individuals).

### Sociodemographic characteristics

In order to obtain the sociodemographic information, we used the standard questionnaire from the WHO MONICA project.^17,18^ The socio-economic status was determined as proposed by the Brazilian Institute of Geography and Statistics and published elsewhere,^19,20^ according to a score obtained using a questionnaire completed during the home visit, in which individuals were classified into one of five levels (A, B, C, D or E). The A stratum represents the highest social class and E the lowest. In view of the small number of subjects in class E, classes D and E were combined for the purpose of this study and to preserve the power of the study. The construction of the score took into consideration family monthly income (class A ≥15 minimum salary and class E ≤1 minimum salary; the Brazilian monthly minimum salary is US$300), education level and number of consumer goods available at home (television, refrigerator, freezer, automobiles etc.). The education levels of participants were classified into three categories according to the number of years attending school: high (completed higher education or higher certified technicians, ≥12 y), intermediate (completed high school, 8–12 y) and low (elementary school or lower, <8 y).

### Statistical analyses

Statistical analyses were performed using SPSS version 22 (IBM, Armonk, NY, USA). Data are presented as mean±SD for continuous variables or as frequency and percentage for dichotomous variables. The overall adequacy for the Gaussian distribution was assessed by the Kolmogorov–Smirnov test. Before each test we checked for correlation and multicollinearity. All analyses were stratified by sex to avoid biological differences between men and women in the lipid profile and also in the response to socio-economic status and education level.

Student’s *t*-test was used to evaluate differences between two independent means. Analysis of variance was used to evaluate differences between three or more means, and in case of a significant F test, Tukey’s post hoc test was used to identify specific differences. Analysis of covariance was used to test the association between blood lipids within the socio-economic and educational strata. Proportions were compared using the χ^2^ or Cochran–Mantel–Haenszel test for trend as appropriate. The association between dyslipidemia and its determinants was evaluated through a multiple logistic regression, controlling for all variables included in the model. A multinomial logistic regression was used to assess the association between socio-economic status and the number of altered lipid variables in men and women after pertinent adjustments for age, BMI, education level and hypertension. For that analysis, we categorized individuals by their number of altered specific blood lipids (total cholesterol, LDL-c, HDL-c and TG). The statistical significance was set at p<0.05 for proportions and means.

**Table 1:** Clinical and anthropometric characteristics of participants stratified by sex

Characteristics	Men (n=737)	Women (n=877)	p-Value	All (n=1614)
Age (y)	44.4±10.8	44.5±10.7	1	44.5± 0.7
Weight (kg)	74.3±13.1	65.4±14.4	<0.0001	69.5±14.5
Height (cm)	169.3±7.1	156.8±6.2	<0.0001	162.6±9.1
BMI (kg/m^2^)	25.9±4.0	26.6±5.5	0.0038	26.2±4.9
WC (cm)	89.0±10.9	83.6±12.9	<0.0001	86.1±12.3
WHR	0.92±0.07	0.84±0.08	<0.0001	0.87±0.09
Fasting glucose (mg/dL)	105.1±27. 2	103.3±32.8	0.2321	104.1±30.4
Cholesterol (mg/dL)	211.7±44.0	214.6±44.7	0.554	213.3±44.4
HDL-c (mg/dL)	42.3±12.4	48.0±12.0	<0.0001	45.4±12.5
LDL-c (mg/dL)	140.1±38.6	143.5±39.3	0.1121	142.0±39.0
VLDL-c (mg/dL)	28.4±16.9	22.7±15.8	<0.0001	25.3±16.2
TG (mg/dL)	152.0±102.9	117.3±83.1	<0.0001	133.1±94.2
Uric acid (mg/dL)	5.5±1.4	4.2±1.3	<0.0001	4.8±1.5
SBP (mmHg)	130.1±19.7	125.8±23.3	<0.0001	127.8±21.8
DBP (mmHg)	87.1±14.0	81.9±13.9	<0.0001	84.3±14.1
HR (bpm)	68.7±10.2	72.2±11.4	<0.0001	70.8±11.1
Hypertension, n (%)	354 (47.0)	328 (37.3)	<0.0001	682 (42.2)
Diabetes, n (%)	55 (7.4)	68 (7.7)	0.9003	123 (7.6)
Overweight/obese, n (%)	418 (56.6)	475 (54.4)	0.3281	893 (55.3)

Data are presented as mean±SD. BMI: body mass index; HR: heart rate; VLDL-c: very low-density lipoprotein cholesterol; WC: waist circumference; WHR: waist-to-hip ratio.

## Results

### Clinical and anthropometric characteristics

Table 1 shows the clinical and anthropometric characteristics of all individuals included in the study, stratified by sex. The final sample for this analysis comprised 1614 participants (mean age in men 44.4±10.8 y vs 44.5±10.7 in women) without lipid-lowering medication or on a cholesterol-restricted diet. BMI was higher among women, while waist circumference and weight:height ratio were higher in men. Cholesterol levels were similar among men and women (211.7±44.0 in men vs 214.6±44.7 y in women; p>0.05), while HDL-c levels were higher in women (42.3±12.4 in men vs 48.0±12.0 in women; p<0.05). Other clinical and anthropometric variables are displayed in Table 1.

### Lipid profile and socio-economic and education levels

Socio-economic and educational characteristics of participants stratified by sex are displayed in Supplementary Table S1. We observed that 57.4% of our sample presented a low education level and 63.2% were included in the lower socio-economic strata (C and D+E). No differences between sexes were observed for socio-economic and education levels (Supplementary Table S1).

**Figure 1:**
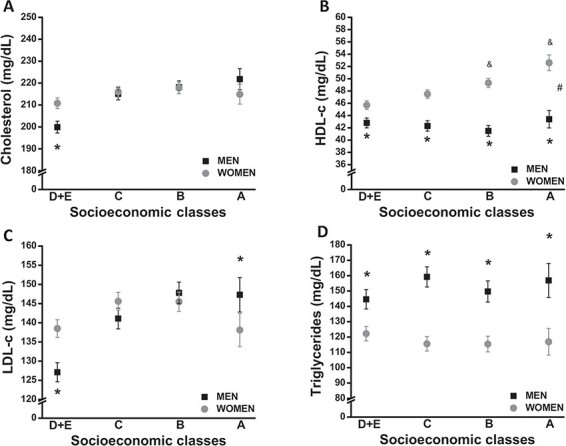
Lipid profile based on socio-economic stratum in men and women. Crude and adjusted (age, BMI, hypertension, smoking and status of physical activity) levels of (A) total cholesterol, (B) HDL-c, (C) LDL-c and (D) TG are presented according to the socio-economic classes. Data are presented as mean±SEM. *p<0.05 between sexes at a specific point, ^#^p<0.05 for interaction, ^&^p<0.05 vs DE category.

**Figure 2:**
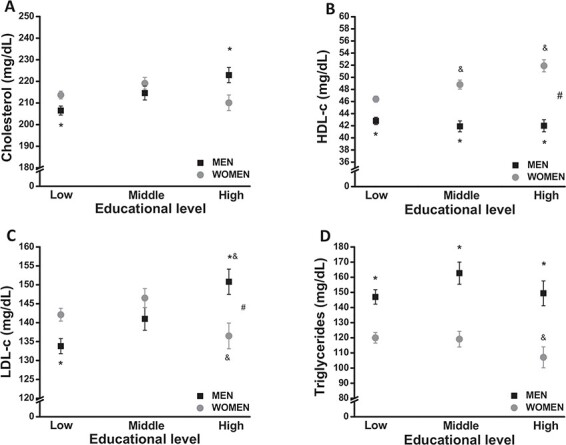
Lipid profile based on education levels in men and women. Crude and adjusted (age, BMI, hypertension, smoking and status of physical activity) levels of (A) total cholesterol, (B) HDL-c, (C) LDL-c and (D) TG are presented according to the educational categories. Data are presented as mean±SEM. *p<0.05 between sexes at a specific point, ^#^p<0.05 for interaction, ^&^p<0.05 vs low category.

Total cholesterol, HDL-c, LDL-c and TG levels between men and women stratified by the different socio-economic and education levels are displayed in Figures 1 and 2, respectively. When we evaluated the lipid profile in relation to socio-economic status, a different pattern between men and women was observed. In men, the higher the socio-economic status, the higher the total cholesterol, LDL-c and TG levels and the lower the HDL-c level. These results show the same pattern after adjustments for age, BMI, hypertension, smoking and physical activity status (Figure 1). Among women, total cholesterol and HDL-c levels increase as the socio-economic status goes from the lowest to the highest. Nevertheless, although the LDL-c level increases as socio-economic status improves, its level declines for the upper class, returning to the same level as in the lower socio-economic strata. On the other hand, the TG level decreases from the lower to the middle strata, remaining stable to the upper strata. There were no significant changes in any factor when controlling for age, BMI and physical activity status (Figure 1). Even when socio-economic status was stratified into three categories (AB, C and DE), the results were the same (data not shown).

When the lipid profile was evaluated by the education level in men, we observed that the higher the education level, the higher the cholesterol, LDL-c and TG levels and the lower the HDL-c level. Those results remained unchanged even after adjustments for age, BMI, hypertension, smoking and physical activity status (Figure 2). Among women, however, results were the opposite. We observed that the higher the education level, the lower the total cholesterol, LDL-c and TG levels and the higher the HDL-c level. Those results were maintained even after adjustments for age, BMI and physical activity status (Figure 2).

### Prevalence of dyslipidemia and related factors

Supplementary Table S2 shows the proportion of alterations in total cholesterol, HDL-c, LDL-c and TG levels according to education level and socio-economic status. In men, the frequency of high total cholesterol, LDL-c and TG increases as they rise from the lower to the upper socio-economic and educational strata. There was a nonsignificant trend to increase HDL-c levels over the socio-economic strata. In women, however, the frequency of lower HDL-c decreases from the lowest to the highest socio-economic and educational strata.

**Figure 3:**
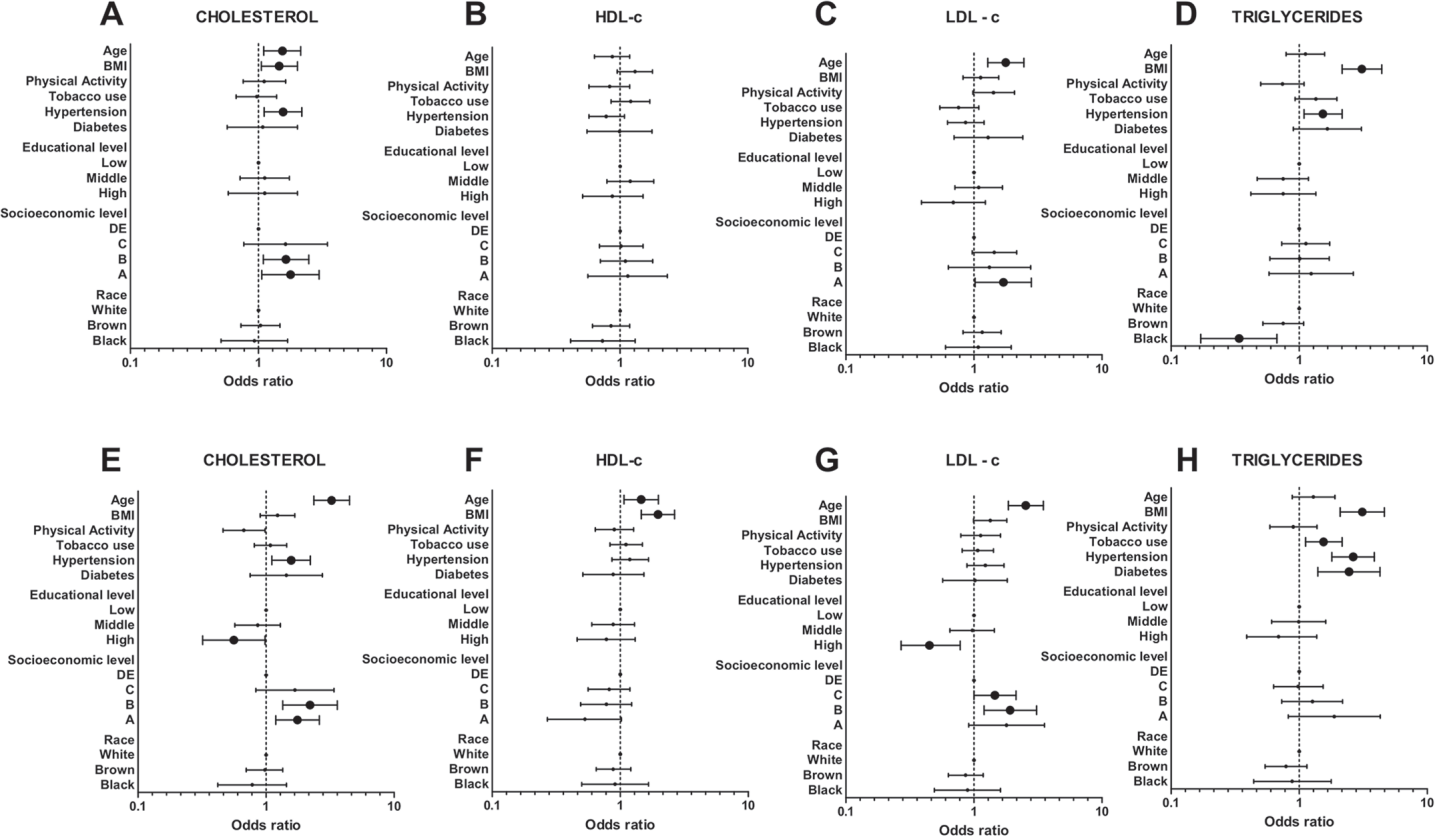
Logistic regression for the association between categorized lipid profile and risk factors in (A–D) men and (E–H) women. Significant associations are highlighted with bigger symbols. Data represent adjusted OR and 95% CI.

Figure 3 shows the adjusted logistic regression for the association between dyslipidemia and potential risk factors, stratified by sex. Age (OR 1.54 [95% CI 1.10 to 2.14]), BMI (OR 1.45 [95% CI 1.05 to 2.02]) and hypertension (OR 1.56 [95% CI 1.11 to 2.18]) were significantly associated with a higher prevalence of hypercholesterolemia in men, but only age (OR 3.25 [95% CI 2.36 to 4.48]) and hypertension (OR 1.57 [95% CI 1.11 to 2.22]) were associated in women. As observed, education levels were not associated with dyslipidemia (higher total cholesterol, LDL-c and TG or lower HDL-c) in men. In women, however, higher education level was associated with lower odds of developing a higher total cholesterol (OR 0.56 [95% CI 0.32 to 0.98]) or LDL-c level (OR 0.45 [95% CI 0.27 to 0.78]) (Figure 3E and G). Also, higher socio-economic strata were significantly associated with elevated total cholesterol in men (OR 1.78 [95% CI 1.06 to 2.98]) and women (OR 1.76 [95% CI 1.19 to 2.60]) and LDL-c (OR 1.70 [95% CI 1.02 to 2.81]) in men.

We also stratified participants by their number of altered parameters on the lipid profile as a function of their socio-economic status. As shown in Table 2, the association between higher socio-economic status and dyslipidemia gets stronger as the number of altered factors increases, being approximately three times higher for men (OR 2.99 [95% CI 1.23 to 5.07]) and approximately two times higher for women (OR 2.31 [95% CI 1.09 to 5.83]) in the A class as compared with their counterparts in class D+E, even after controlling for age, BMI, education level and hypertension. The results did not change when socio-economic status was classified in three categories (AB, C and DE) (data not shown).

## Discussion

Coronary heart disease is the main cause of death in developed countries.^2^ Some common risk factors are strongly associated with the development of coronary heart disease. One of the leading risk factors is dyslipidemia, which has been proven to be directly associated with the risk of myocardial infarction.^21,22^ Dyslipidemia is a complex disorder influenced by several factors related to genetic background and multiple environmental factors.^23^ Dyslipidemia is associated with inadequate nutrition and low levels of physical activity, but also socio-economic disparities.

A significant number of studies performed in developed countries of North America and Europe have shown an inverse relationship between socio-economic status and the risk of developing CVDs.^24,25^ This relationship was explained by McCurley et al.,^26^ who showed that people of a higher socio-economic status have a reduced risk of dyslipidemia through the mediation action of psychosocial factors (e.g. anxiety, depression and social support). However, when evaluating studies performed in developing countries, research on the relationship between socio-economic status and the risk for CVDs is relatively scarce and the findings are less consistent.^27^ In fact, some studies have demonstrated a positive association between increased blood lipids and socio-economic status. In our study, we observed a significant positive association between socio-economic status and dyslipidemia, in which total cholesterol and LDL-c were increased in both men and women in the higher socio-economic strata, even after controlling for confounders. Also, higher socio-economic status was associated with an increased prevalence of hypercholesterolemia, regardless of sex. Our data corroborates a previous report by Sun et al.,^28^ in which dyslipidemia was positively associated with socio-economic status in China. Also, dyslipidemia was more prevalent in individuals of higher socio-economic status in urban regions of India.^29^ However, authors studying populations from developed countries were unable to prove such an association.^30^

A possible explanation for this positive association between dyslipidemia and socio-economic status lies in the fact that social improvement facilitates easy access to some attractive unhealthy behaviors (fast food-based diet, physical inactivity, and alcohol and tobacco use). On the other hand, this effect seems to not be explained by or related to psychosocial factors. This statement corroborates a study of middle-aged Mexican American women in which psychosocial factors were not able to explain the association between socio-economic status and metabolic parameters such as dyslipidemia.^31^ Another important finding of the present study was that socio-economic status was independently associated with dyslipidemia regardless of classical risk factors such as age, BMI, physical activity, hypertension, smoking and education level.

Some of the associations between lipid profile and socio-economic status observed in our study were different between men and women. For instance, we observed that HDL-c and TG levels increased in women from the lower to the higher socio-economic strata, while in men they remained steady throughout the socio-economic categories. These sex differences in the association between socio-economic status and dyslipidemia have been reported by authors elsewhere. Using data from the European Prospective Investigation of Cancer (EPIC) – Norfolk Study, Shohaimi et al.^5^ showed that the association between lipid levels and socio-economic indicators were more evident in women than in men. The authors showed that women in lower socio-economic strata had higher levels of LDL-c and TG. Corroborating the sex differences reported by Shohaimi et al.,^5^ a study conducted in South Korea showed a prevalence of dyslipidemia of 46.8% in men and 31% in women.

**Table 2:** Multinomial logistic regression for the association between socio-economic status and the number of components of dyslipidemia in a population-based study

Characteristics	Socio-economic status, adjusted OR* (95% CI)			
	D+E	C	B	A
Men	–	–	–	–
No dyslipidemia	1 (reference)	1 (reference)	1 (reference)	1 (reference)
1 factor for dyslipidemia	1 (reference)	1.34 (0.71 to 2.55)	0.85 (0.49 to 1.99)	1.12 (0.43 to 5.17)
2 factors for dyslipidemia	1 (reference)	1.33 (0.73 to 2.42)	1.15 (0.55 to 2.79)	1.34 (0.78 to 2.72)
3 factors for dyslipidemia	1 (reference)	1.65 (0.85 to 3.12)	1.12 (0.53 to 2.92)	1.52 (0.88 to 3.49)
4 factors for dyslipidemia	1 (reference)	2.32 (1.02 to 5.25)	2.26 (1.01 to 5.04)	2.99 (1.23 to 5.07)
Women	–	–	–	–
No dyslipidemia	1 (reference)	1 (reference)	1 (reference)	1 (reference)
1 factor for dyslipidemia	1 (reference)	0.79 (0.42 to 1.58)	1.04 (0.57 to 2.33)	1.15 (0.67 to 2.58)
2 factors for dyslipidemia	1 (reference)	1.75 (0.78 to 4.99)	1.67 (0.80 to 3.82)	2.02 (0.89 to 5.63)
3 factors for dyslipidemia	1 (reference)	1.21 (0.54 to 3.35)	1.66 (0.89 to 3.45)	1.87 (0.90 to 4.13)
4 factors for dyslipidemia	1 (reference)	1.76 (0.60 to 5.13)	2.40 (1.17 to 5.31)	2.31 (1.09 to 5.83)

We divided participants into five categories based on the number of altered blood lipids.

*Adjusted for age, BMI, education level, smoking and hypertension. We only consider total cholesterol, HDL-c, LDL-c and TG as factors for dyslipidemia.

Although socio-economic status and education level might be related to each other at some point (once the education level is part of the socio-economic construct), we observed different patterns of changes in the blood lipid profile by using either the socio-economic construct or the education level separately. When evaluated independent of the socio-economic status, education level has been significantly associated with changes in the blood lipid profile. For instance, young adults with higher education levels had higher levels of total cholesterol and TG than those with lower education levels.^32^ In a rural region of China, a general inverse association was reported between education level and dyslipidemia.^28^ Similar data were observed with Greek adolescents.^33^ Also, evaluating the Greek participants of the EPIC Study, Benetou et al.^8^ observed that total blood cholesterol was inversely related to education level in both men and women. Our results partially corroborate Benetou et al.’s report, that the higher the education level, the lower the total cholesterol, LDL-c and TG in women. Moreover, the results were the opposite for men. These data give clear evidence for the important contribution of sex differences in the association of blood lipids and socio-economic and educational status. However, after a full adjustment, education level was not associated with dyslipidemia in men, but was associated with lower odds of developing high total cholesterol and LDL-c levels in women. This association was observed in preschoolers from Brazil, in which Nobre et al.^34^ showed that children whose mothers had a lower educational background were at higher risk of a high LDL-c level.

Our data suggest that education level should not be used as synonymous with socio-economic status. In fact, socio-economic categorization involves different variables, including occupational status, regular family income, house amenities and education level. This idea was supported by Nam et al.,^12^ who found different results when dyslipidemia was associated with socio-economic status or education level. In contrast with the socio-economic levels assessed by the score-based questionnaires we used in our study, education levels per se were not associated with any of the blood lipids tested in our fully adjusted regression model in men, but a higher education level was related to lower total cholesterol and LDL-c in women. These results were the opposite when socio-economic status was used. Also, socio-economic status and education level showed only low collinearity in our study, which may indicate that they have different meanings and these concepts should not be used interchangeably.

Our study has some strengths and limitations. In our study, socio-economic status was defined by a construct of some parameters instead of using only monthly income or education level alone (as a surrogate to socio-economic classification). This fact reinforces the strength of our data as compared with other studies. Also, our analyses were stratified by sex, which gave us important results without the interference of biological differences in the lipid profile between men and women. In addition, we presented sex-specific differences in the lipid profile based on different socio-economic strata. Although we have a large sample size from a general urban population, some analyses using socio-economic status were divided into several categories, which might reduce the ability to generalize our results. However, when analyses were performed using three categories of socio-economic status (instead of four), the results were similar (data not shown).

## Conclusions

In summary, our data show a consistent association between higher socio-economic status and dyslipidemia in a population-based study carried out in an urban area in Brazil. Both men and women in the upper social classes had higher levels of total cholesterol and LDL-c, showing more than a two times higher chance of having multiple alterations in the lipid profile. These results point to the need for screening and primary care in developing countries based on literacy, to minimize the health problems associated with socio-economic status. It also calls attention to the fact that those individuals who have the ability to pay for healthy foods are the ones at higher risk for dyslipidemia.

## Supplementary Material

ihz089_Supplementary_Tables_R2
