# Supplementary material for: Socioeconomic status and education level are associated with dyslipidemia in adults not taking lipid-lowering medication: a population-based study
Source: Int Health. 2019 Nov 6;14(4):346–53. doi: 10.1093/inthealth/ihz089 (PMC10575599; doi:10.1093/inthealth/ihz089)
Supplement: ihz089_Supplementary_Tables_R2 [file ihz089_supplementary_tables_r2.docx]

**Supplementary Table S1.** Socioeconomic and educational characteristic of participants stratified by sex.

|  | **MEN** | **WOMEN** | ***P* Value** | **TOTAL** |
| --- | --- | --- | --- | --- |
| **Educational level**  ***Low***  ***Intermediate***  ***High***  **Socioeconomic status**  ***D+E***  ***C***  ***B***  ***A*** | 407 (56.1%)  177 (24.4%)  141 (19.4%)  242 (33.0%)  218 (29.7%)  196 (26.7%)  77 (10.5%) | 497 (58.5%)  228 (26.8%)  125 (14.7%)  277 (31.9%)  276 (31.8%)  234 (26.9%)  82 (9.4%) | 0.058  0.801 | 904 (57.4%)  405 (25.7%)  266 (16.9%)  519 (32.4%)  494 (30.8%)  430 (26.8%)  159 (9.9%) |

Data are shown as frequency (and percentage).

**Supplementary Table S2.** Frequency of dyslipidemia in men and women stratified by socioeconomic status and educational level

|  | **MEN** | | | |  | **WOMEN** | | | |
| --- | --- | --- | --- | --- | --- | --- | --- | --- | --- |
|  | **Cholesterol**  **(**≥200 mg/dL) | **HDL-c**  **(**<40 mg/dL) | **LDL-c**  **(**≥160 mg/dL) | **Triglycerides**  **(**≥150 mg/dL) |  | **Cholesterol**  **(**≥200 mg/dL) | **HDL-c**  **(**<50 mg/dL) | **LDL-c**  **(**≥160 mg/dL) | **Triglycerides**  **(**≥150 mg/dL) |
| **Socioeconomic status**  ***D+E (lowest)***  ***C***  ***B***  ***A (highest)***  ***P for trend***  **Educational level**  ***Low***  ***Intermediate***  ***High***  ***P for trend*** | 116/243 (47.9%)  132/221 (60.5%)  128/202 (64.3%)  53/79 (67.5%)  <0.0001  -  224/441 (54.9%)  106/180 (59.9%)  94/144 (65.7%)  0.0206 | 106/243 (45.7%)  101/221 (49.0%)  94/202 (49.2%)  40/79 (52.0%)  0.2473  -  184/441 (47.7%)  76/180 (44.7%)  74/144 (52.8%)  0.4418 | 113/243 (48.5%)  124/221 (60.2%)  129/202 (67.5%)  52/79 (68.0%)  <0.0001  -  216/441 (55.8%)  99/180 (58.2%)  98/144 (70.0%)  0.0059 | 78/243 (32.4%)  87/221 (40.1%)  79/202 (39.9%)  39/79 (49.3%)  0.0072  -  142/441 (35.9%)  73/180 (41.2%)  62/144 (43.7%)  0.0434 |  | 151/279 (54.1%)  169/277 (61.0%)  154/237 (65.0%)  50/84 (59.5%)  0.0503  -  308/504 (61.1%)  138/232 (59.5%)  72/132 (54.5%)  0.1911 | 187/279 (68.7%)  171/277 (62.7%)  132/237 (52.6%)  37/84 (45.1%)  <0.0001  -  328/504 (66.5%)  131/232 (56.9%)  64/132 (49.6%)  0.0001 | 151/279 (55.5%)  163/277 (59.9%)  150/237 (64.1%)  49/84 (59.7%)  0.1190  -  300/504 (60.8%)  141/232 (61.6%)  66/132 (51.2%)  0.1058 | 72/279 (23.8%)  60/277 (21.5%)  51/237 (21.5%)  20/84 (23.8%)  0.4039  -  131/504 (26.0%)  46/232 (19.8%)  24/132 (18.2%)  0.0235 |

Data are shown as frequency of dyslipidemia/total sample for each category (and percentage).
